# Implementation and effectiveness of a physician-focused peer support program

**DOI:** 10.1371/journal.pone.0292917

**Published:** 2023-11-01

**Authors:** Molly L. Tolins, Jamal S. Rana, Suzanne Lippert, Christopher LeMaster, Yusuke F. Kimura, Dana R. Sax

**Affiliations:** 1 Department of Emergency Medicine, Kaiser Permanente East Bay, The Permanente Medical Group, Oakland, California, United States of America; 2 Department of Cardiology, Kaiser Permanente Oakland Medical Center, The Permanente Medical Group, Oakland, California, United States of America; 3 Division of Research, Kaiser Permanente Northern California, Oakland, California, United States of America; University of Oxford, UNITED KINGDOM

## Abstract

**Background:**

The practice of medicine faces a mounting burnout crisis. Physician burnout leads to worse mental health outcomes, provider turnover, and decreased quality of care. Peer support, a viable strategy to combat burnout, has been shown to be well received by physicians.

**Methods:**

This study evaluates the Peer Outreach Support Team (POST) program, a physician-focused peer support initiative established in a 2-hospital system, using descriptive statistical methodologies. We evaluate the POST program using the Practical Robust Implementation and Sustainability Model (PRISM) framework to describe important contextual factors including characteristics of the intervention, recipients, implementation and sustainability infrastructure, and external environment, and to assess RE-AIM outcomes including reach, effectiveness, adoption, implementation, and maintenance.

**Results:**

This program successfully trained 59 peer supporters across 11 departments in a 2-hospital system over a 3-year period. Trained supporters unanimously felt the training was useful and aided in general departmental culture shift (100% of respondents). After 3 years, 48.5% of physician survey respondents across 5 active departments had had a peer support interaction, with 306 successful interactions recorded. The rate of interactions increased over the 3-year study period, and the program was adopted by 11 departments, representing approximately 60% of all physicians in the 2-hospital system. Important implementation barriers and facilitators were identified. Physician recipients of peer support reported improved well-being, decreased negative emotions and stigma, and perceived positive cultural changes within their departments.

**Conclusions:**

We found that POST, a physician-focused peer support program, had widespread reach and a positive effect on perceived physician well-being and departmental culture. This analysis outlines a viable approach to support physicians and suggests future studies considering direct effectiveness measures and programmatic adaptations. Our findings can inform and guide other healthcare systems striving to establish peer support initiatives to improve physician well-being.

## Introduction

Burnout is an existential threat to the field of medicine. Burnout and secondary trauma erode providers’ competency, physical and mental health, and morale [1–3]. The harm to clinicians contributes to systemic harm and is associated with worse patient outcomes [4], more frequent medical errors [5], and higher provider turnover [6]. The COVID-19 pandemic has amplified these challenges [7–10]. Nurse and physician shortages–whether due to COVID sick leave or from leaving the profession altogether–compound the harms for the providers that remain [6,11,12].

Perfectionism and mental toughness–reinforced during medical school and residency–pervade the culture of medicine. Emotional reactions to adverse events are seldom acknowledged or discussed. Physicians may feel unable to ask for help, leaving them isolated and ashamed [13]. Many healthcare organizations lack a formal support structure for affected physicians [14]. Even in those that do, many providers fear stigma from a mental health diagnosis or the possibility that seeking help will hinder career advancement [15,16]. There is a need to create programs to support personal and systemic resilience and provide psychological first aid to physicians and other healthcare workers in a confidential, safe, non-judgmental space [17].

Commonly recommended strategies rely on the individual to perform self-care or seek mental health resources [6]: “*Medice*, *cura te ipsum*.” However, stigma, isolation, and time constraints pose significant barriers to physicians accessing self-care resources after a traumatic incident [17]. Proactive, confidential peer support may be especially useful for those who are most isolated and at risk. The opportunity to meet confidentially with trained peers can create alternate, more acceptable healing pathways than traditional approaches such as Employee Assistance Programs (EAPs) or psychological counseling [18,19].

We describe a novel physician-focused peer support program developed within Kaiser Permanente Northern California (KPNC). While previously described healthcare and physician peer support programs have been studied, the POST model is distinct in its focus on physicians, a robust third-party referral structure, a hyperlocal framework, and strong institutional support.

Previously described models of peer support programs in healthcare have reported relatively low physician utilization [20–23], with most programs offering support to both physicians and non-physician staff, and barriers shown to include time constraints, a culture of toughness, stigma, and access. To our knowledge, none of the studied peer support programs established worldwide have offered paid time for this work to both the supporter and supported physician which we hypothesize will help to surmount these barriers. Most studied programs offer a hospital-wide team of supporters; because research has shown physicians are most likely to accept and benefit from support from peers [19], we have intentionally chosen to provide peer-to-peer matching at the departmental and specialty level. While previous studies have quantified referrals or outreaches, we intentionally report only successful interactions, allowing for a more pragmatic description of program reach and maintenance. Further, to our knowledge, this is the first study to incorporate broad departmental physician end-user feedback, allowing for an evaluation of perceived effectiveness and identification of important implementation facilitators and barriers not previously described. This program has institutional support and provides robust training for peer physicians, equipping them to engage in a proactive, department-based support program.

Herein we apply descriptive statistical methodologies to evaluate the POST program using the Practical Robust Implementation and Sustainability Model (PRISM) framework [24]. Our primary objective is to provide a comprehensive description and evaluation of POST, a physician-focused peer support program. In so doing, we hope to establish guiding principles for creating similar programs elsewhere to best support frontline physicians in these unprecedented times.

## Methods

The Practical Robust Implementation and Sustainability Model (PRISM) was developed to provide a practical, actionable model that could be used to plan and guide interventions and their implementation [24]. PRISM is a model that encompasses the widely used Reach, Effectiveness, Adoption, Implementation, Maintenance (RE-AIM) evaluative framework [25]. PRISM proposes that key contextual factors influence the RE-AIM outcomes, including implementation and sustainability infrastructure and external environment factors. We selected PRISM as an evaluative framework because it identifies important contextual factors (which facilitate planning and implementation and guide purposeful adaptations) while evaluating relevant RE-AIM outcomes [24–26].

**Fig 1:**
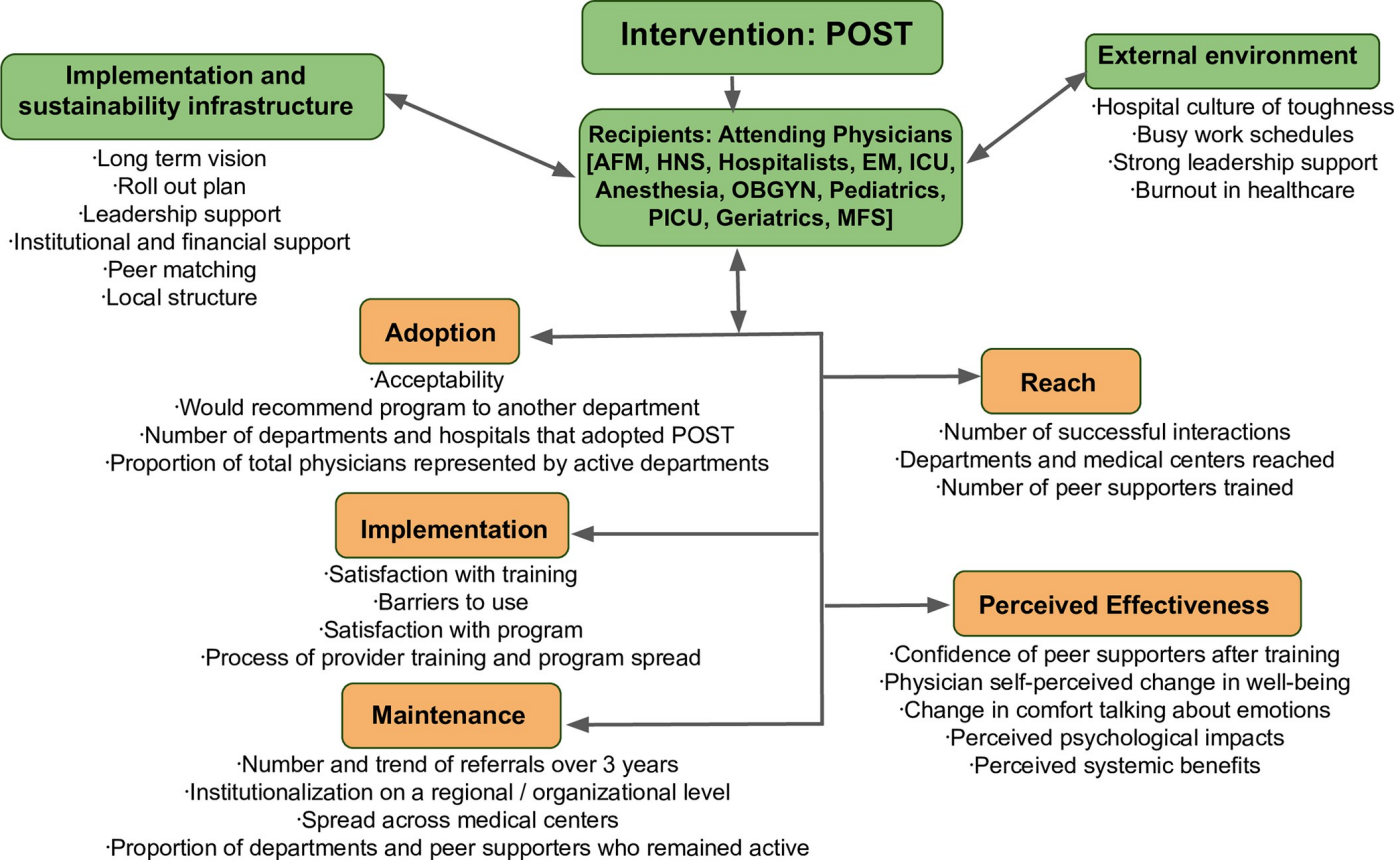
The Practical Robust Implementation and Sustainability Model (PRISM) as applied to POST. In green at the top are PRISM factors, including the domains of intervention, recipients, implementation and sustainability infrastructure, and external environment, with important components included. Below in orange are the RE-AIM outcomes including reach, perceived effectiveness, adoption, implementation and maintenance. Included below these outcomes are major considerations for each. AFM, adult and family medicine; HNS, head and neck surgery; EM, emergency medicine; ICU, intensive care unit; OBGYN, obstetrics and gynecology; PICU, pediatric ICU; MFS, maxillofacial surgery.

Applying the PRISM framework, we describe the intervention, the recipients of the intervention, implementation and sustainability infrastructure, and the external environment (PRISM factors), and evaluate reach, effectiveness, adoption, implementation, and maintenance (RE-AIM outcomes). Fig 1 includes an overview of our application of the PRISM framework.

### PRISM domains

#### Intervention

A group of KPNC physician leaders and key stakeholders–front-line providers in high-risk specialties, operational leads, and experts from medical-legal, human resources, patient safety, and physician wellness departments–convened to address the gap in support for physicians. This group designed the Peer Outreach Support Team (POST) program drawing from literature review, psychological first-aid education, applied educational pedagogy, and expert opinion. Prior peer support programs had limited physician reach, institutional support, peer matching, and end-user evaluation, and these were barriers to the effectiveness of these programs. The POST program was purposefully designed to address these limitations. As we describe below, POST is unique among organizational peer support programs in its system of third-party referrals, robust peer supporter training, institutional support, and physician focus and reach.

The stated purpose of POST is to leverage the shared experience of trained peers to provide physicians with connection and support for professional stress of any kind.

**Fig 2:**
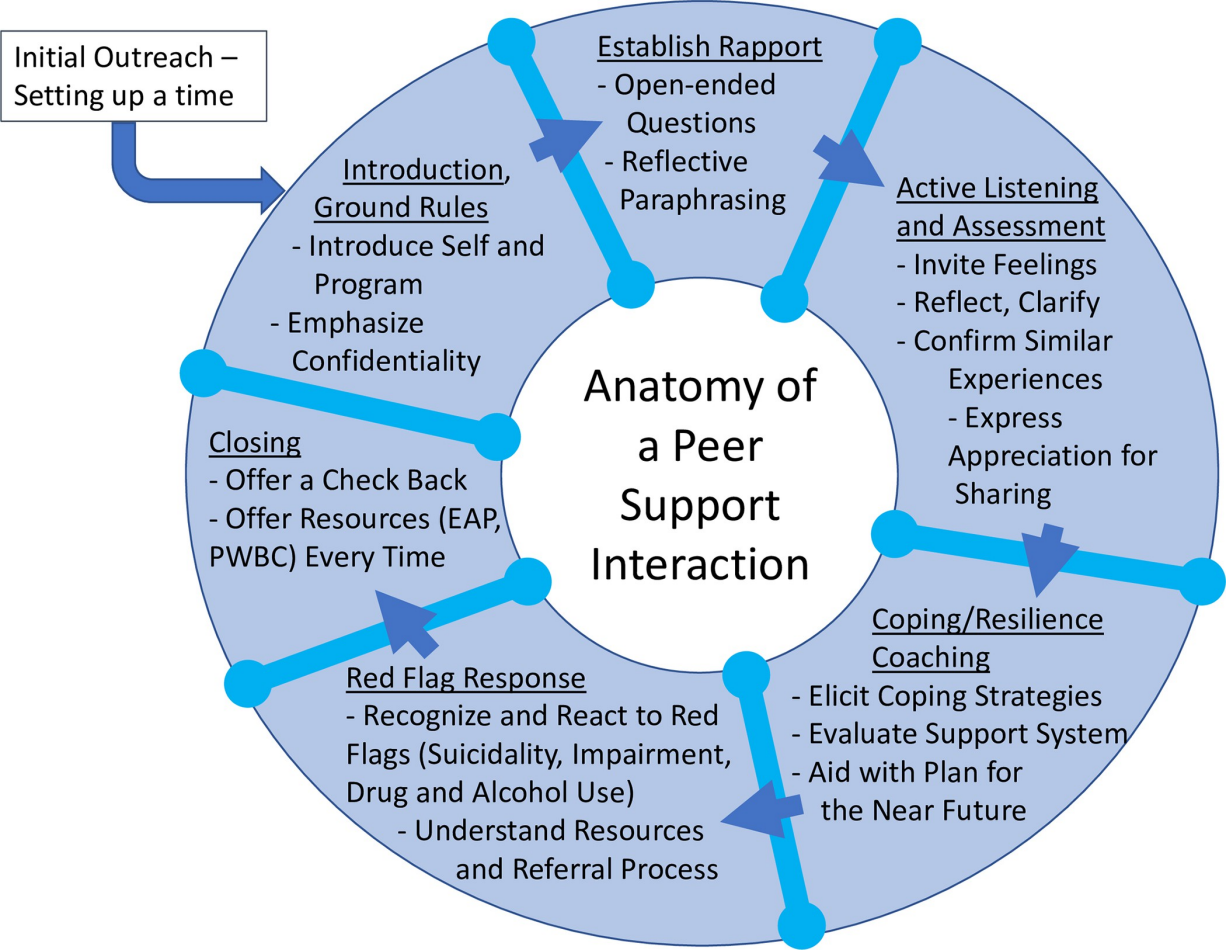
The anatomy of a peer support interaction. Overview of the components emphasized in the 4-hour training starting from the initial outreach and proceeding to the closing of the interaction. Tools and scripting are provided for each of these components within the training. EAP, Employee Assistance Program; PWBC, Physician Well Being Committee.

All peer supporters undergo an initial 4-hour training including: understanding of burnout and peer support; confidentiality and risk concerns; program structure; the components of a peer support interaction (Fig 2); identification of red flags and how to respond; how to access additional resources; and hands-on practice. These components were designed with consideration of both local needs and best practices from external programs [23,27,28]. Initially administered in person, the POST training transitioned to a virtual platform with the start of the COVID-19 pandemic, allowing for safe social distancing, ease of breakout rooms and hands-on practice, and limited need for administrative support, as no physical space or materials are required for this virtual training.

To protect peers and the institution in the case of future legal action, the training emphasizes the importance of maintaining confidentiality, avoiding discussion of case details, and pivoting towards emotional support, with scripting and practice provided. Like previous programs, local legal advisors agreed that peer support poses a very low risk of legal harm and offers crucial benefits for both physicians and patients [23].

#### Recipients

The program was initiated in 2 hospitals staffed by a shared physician group in June of 2019 and has continued to spread since then. See S1 and S2 Files for a timeline of program genesis, provider training, and spread. During the study period, POST was available to attending physicians in the following 11 departments: adult and family medicine (AFM), head and neck surgery (HNS), hospitalists, emergency medicine, intensive care unit (ICU), anesthesia, obstetrics and gynecology (OBGYN), pediatric hospitalists, pediatric ICU (PICU), geriatrics, and maxillofacial surgery (MFS). These physicians are part of the Permanente Medical Group, a group that provides care to patients within KPNC, a large integrated healthcare delivery system providing comprehensive inpatient, ED, and ambulatory care for over 4 million people.

#### Implementation and sustainability infrastructure

This program has benefited from robust institutional support. Peer supporters are paid for their time in training, regular professional development, and check-ins. Both the peer supporter and the physician being supported can bill time for the support interaction. This arrangement is a tacit acknowledgement that physician well-being is valued at an institutional level and addresses the burden of additional time commitments for peer supporters and front-line providers [19]. Because burnout is extremely costly for a healthcare system [29], we believe this model is financially sound.

Once a department decides to launch POST, the program is promoted via email, posters, and departmental meetings, and encourages both self- and third-party referrals. Because physicians are often reluctant to reach out to support services, this third-party referral expands the reach of the program [23]. Referrals are sent by protected email, secure messaging, or direct conversations to the departmental POST lead, who contacts a peer supporter to reach out to the individual within 48 hours.

The success of POST rests critically on the trust each department vests in its peer supporters. To select the most respected and well-positioned supporters, an anonymous survey is sent to all physicians in a participating department to nominate their peers. Nominated physicians are then offered the position. We have found that a 10:1 ratio of front-line providers to trained peer supporters within departments optimizes capacity matching and potential for culture shift [19].

While POST has strong institutional support and is regionally publicized, its adoption in individual departments and medical centers is voluntary and depends on these departments or hospitals to reach out to our regional team to initiate the steps of starting a program. This process depends on word of mouth and grassroots organizing around an identified need in each local environment. This allows a bottom-up approach which ensures buy-in and investment by local leaders.

#### External environment

This program was implemented in a 2-hospital system with strong leadership support, manifesting as financial and institutional support, leadership promotion of the program, and time protection for POST leads. Environmental barriers included busy work schedules limiting time for POST interactions, and a national physician culture of toughness [13]. Burnout in healthcare, which increased during this study nationally [30], also impacts the external environment, and must be considered a facilitator (representing the need for peer support), as well as a potential barrier (individual burnout limiting willingness to engage in peer support).

### POST program evaluation—RE-AIM outcomes

#### Study population

We evaluate the POST program in two ways and among two populations: 1) we evaluate the POST provider training among trained peer support providers and 2) we evaluate the impact of the POST program among the 11 departments and approximately 530 front-line physicians who had access to the POST program during the study period. We included 3 separate sets of data, with study populations for each enumerated below.

#### Reach

To assess program reach, we consider the number of physicians who completed the initial training to become peer supporters, and the number of successful POST interactions between June 2019 and May 2022. All peer supporters were asked to complete a de-identified record including date and length of each completed peer support interaction. If the support was declined, no record was completed. These records were initially kept on paper and the paper form did not collect length of encounter; starting in November 2020 this record-keeping form was transitioned to Microsoft Forms, and length of encounter was captured, thus average time spent was based on interactions recorded via the Microsoft Forms record keeping form between November 2020 –May 2022.

We analyzed all recorded encounters from June of 2019 through April 2022. Descriptive statistics were used to summarize encounter data, including the number of interactions overall and by department, and the average time spent per interaction.

#### Perceived effectiveness

To evaluate effectiveness of the POST program, we considered perceived effectiveness outcomes using peer supporter and end-user physician feedback. To evaluate the training, an anonymous feedback survey (Appendix, Survey 1) was administered to all peer supporters who completed the virtual training from 9/2020-1/2022. These included 38 physicians from the following departments: Anesthesia (1), Intensive Care Unit (ICU) (3), Emergency Medicine (EM) (2), Head and Neck Surgery (1), Maxillofacial Surgery (1), Hospitalists (8), Geriatrics (2), Pediatric Hospitalists (2), Pediatric ICU (1), and Adult and Family Medicine (17). The survey included both categorical and open-ended questions designed to probe physicians’ overall impression of the training, confidence in navigating resources, perceived tool and knowledge acquisition, understanding of the referral process, and perception of the online training platform. The survey content was based on review of successes and limitations of earlier peer support programs.

Descriptive statistics were used to summarize categorical variables. Content analysis was conducted for the open-ended responses. A team of 3 physician-investigators (CL, MT, SL) applied the constant comparative method [31,32], reviewing the open-ended responses iteratively and then jointly developing codes to represent major themes. The team met regularly to discuss discrepancies and refine the coding structure.

To assess the perceived effectiveness of the POST program for front-line physicians, a department-wide anonymous survey was sent to all staff attending physicians in March of 2022 via email in the longest-running departments, including OBGYN, EM, Hospitalists, ICU, and Anesthesia, a total of 255 physicians. Only the longest-running departments, defined as active for 18 months or longer, were included to allow for the accumulation of a meaningful breadth of experience with the program and an adequate number of peer support interactions to speak meaningfully to the interactions themselves. Eighteen months was chosen as a cut off because previous studies have shown a gradual adoption and fewer referrals in the first year [20,23]; based on literature review and our own experience, we believe 18 months allows for adequate momentum, departmental acceptance, and group and individual experience to garner meaningful results. This survey was administered via email to all physicians in these departments regardless of leave or time off status, with a single reminder email 1 week later. The aim of the survey was to assess physicians’ perceptions of the program, including satisfaction, barriers to use, experience with peer support, and perceived impact of the program. The survey tool was developed based on review of peer support literature and was designed to capture how the POST program responded to limitations in these earlier programs. Perceived effectiveness outcomes included physician awareness of the program, self-perceived change in well-being, change in comfort in talking about emotions, and reported departmental culture change. This survey included both categorical and open-ended questions (Appendix, Survey 2). We used similar methods to analyze these surveys as described above.

#### Adoption

We assess adoption at the hospital and health system level by considering the number of departments that adopted the POST program during the study and the number of hospitals who have adopted the program since its launch. We consider the proportion of total physicians in the two-hospital system with active POST teams in their departments. In the department-wide survey, we consider willingness to recommend the program to another department and the proportion who accepted peer support when offered.

#### Implementation

We assess implementation by describing the process of provider training and program spread, guided by the PRISM framework. We also consider satisfaction with the training and the virtual platform, satisfaction with the program, and reported barriers to use.

#### Maintenance

We assess maintenance by reporting the proportion of peer supporters and departments that remained active and the total number and trend in rate over time of POST interactions since the program was launched. We also describe spread to additional medical centers and the institutionalization of the program.

#### Ethical considerations

The Research Determination Committee for the KPNC has determined the project does not meet the regulatory definition of research involving human subjects per 45 CFR 46.102(d). Descriptions of the study and its purpose were included with the anonymous surveys, and participation was optional. All data were analyzed anonymously with no identifying information collected; thus, the informed consent requirement was waived.

## Results

### Reach

A total of 59 physicians underwent the initial 4-hour POST training to become peer supporters between May 2019 and May 2022, with 38 of these completing the virtual training. During the study period, a total of 11 departments launched POST teams.

From June 2019 to May 2022, 306 POST interaction record keeping forms were completed. Of these, the highest utilizer was Emergency Medicine (213, 69.6%), followed by Anesthesia/ICU (49, 16%) and Hospitalists (22, 7.2%). Across the 11 active departments representing approximately 530 physicians, there were an average of 8.5 completed interactions per month over the 36-month period, with a range of 1 to 27, and a median of 8 interactions/month. Three departments had no referrals. The interactions took an average of 68 minutes, with a range of 10–180 minutes and a median of 60 minutes.

Seventy-five physicians responded to the department-wide anonymous survey (out of 255 total physicians, a 29.4% response rate). Table 1 presents sociodemographic and professional characteristics of respondents.

**Table 1:** Survey respondent demographics. Characteristics of front-line physicians who responded to the survey on the impact of POST. EM, Emergency Medicine. OBGYN, Obstetrics and Gynecology. ICU, Intensive Care Unit.

Respondent Demographics (n = 75)	n (%)
Department	
EM	31 (41.3)
Anesthesia	20 (26.7)
OBGYN	15 (20)
ICU	5 (6.7)
Hospitalist	4 (5.3)
Gender	
Men	28 (37.3)
Women	46 (61.3)
Prefer not to say	1 (1.3)
Age (range)	
30–35	6 (8)
35–40	10 (13.3)
40–45	24 (32)
45–50	17 (22.7)
50–55	13 (2.6)
55+	5 (6.7)
Years in Practice (range)	
0–5	6 (8)
5–10	22 (29.3)
10–20	34 (45.3)
20–30	10 (13.3)
30+	3 (4)

Of the 75 respondents, 66 (88%) responded yes to being aware of the POST program. Of these, 30 (45.5%) had made a referral to the program and 16 (24.2%) had made a self-referral. Of those aware of the program, 32 (48.5%) had a POST interaction themselves; 26 (39.4%) had never had a peer supporter reach out; and 8 (12.1%) had had a peer supporter reach out but declined. Of those who had a peer support interaction, 18 (56.3%) had two or more interactions.

### Perceived effectiveness

**Fig 3:**
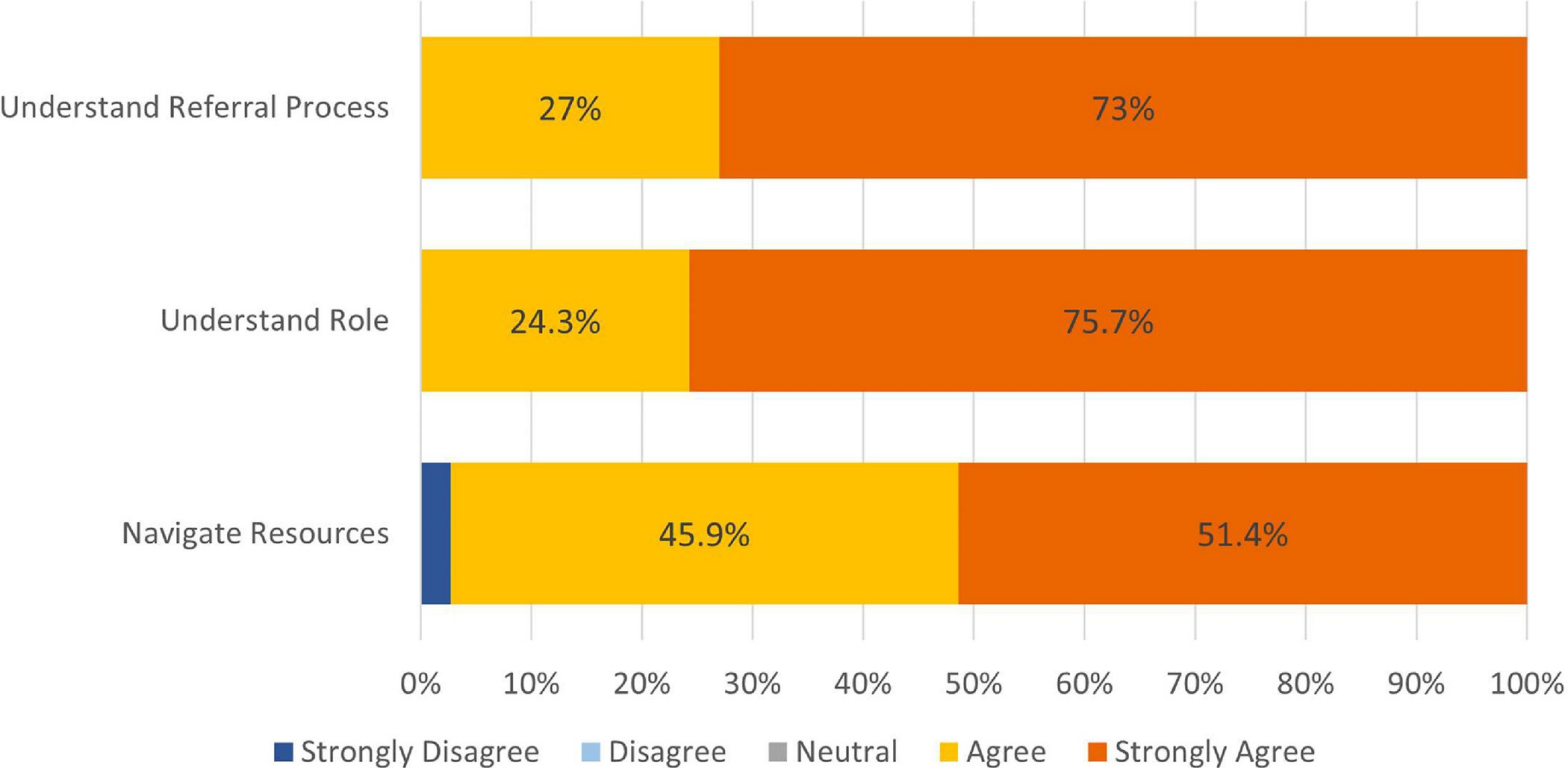
Peer supporter training feedback. Responses to the feedback survey from physicians who underwent the virtual training displayed in stacked percentages. Understanding role refers to role as a peer supporter in the POST program. Resources refers to the Employee Assistance Program, Physician Well Being Committee, and other mental health resources available to physicians after a difficult or adverse event. Number of survey respondents = 37.

Of the 38 trained providers, 37 (97%) responded to the training feedback survey. Of respondents, 100% described the training as good or excellent on a 5-point bimodal Likert scale and 100% agreed that the training gave them a framework and tools to promote a culture of mutual support within their department. Fig 3 shows the proportion of trained providers who felt confident in key components of the training.

**Fig 4:**
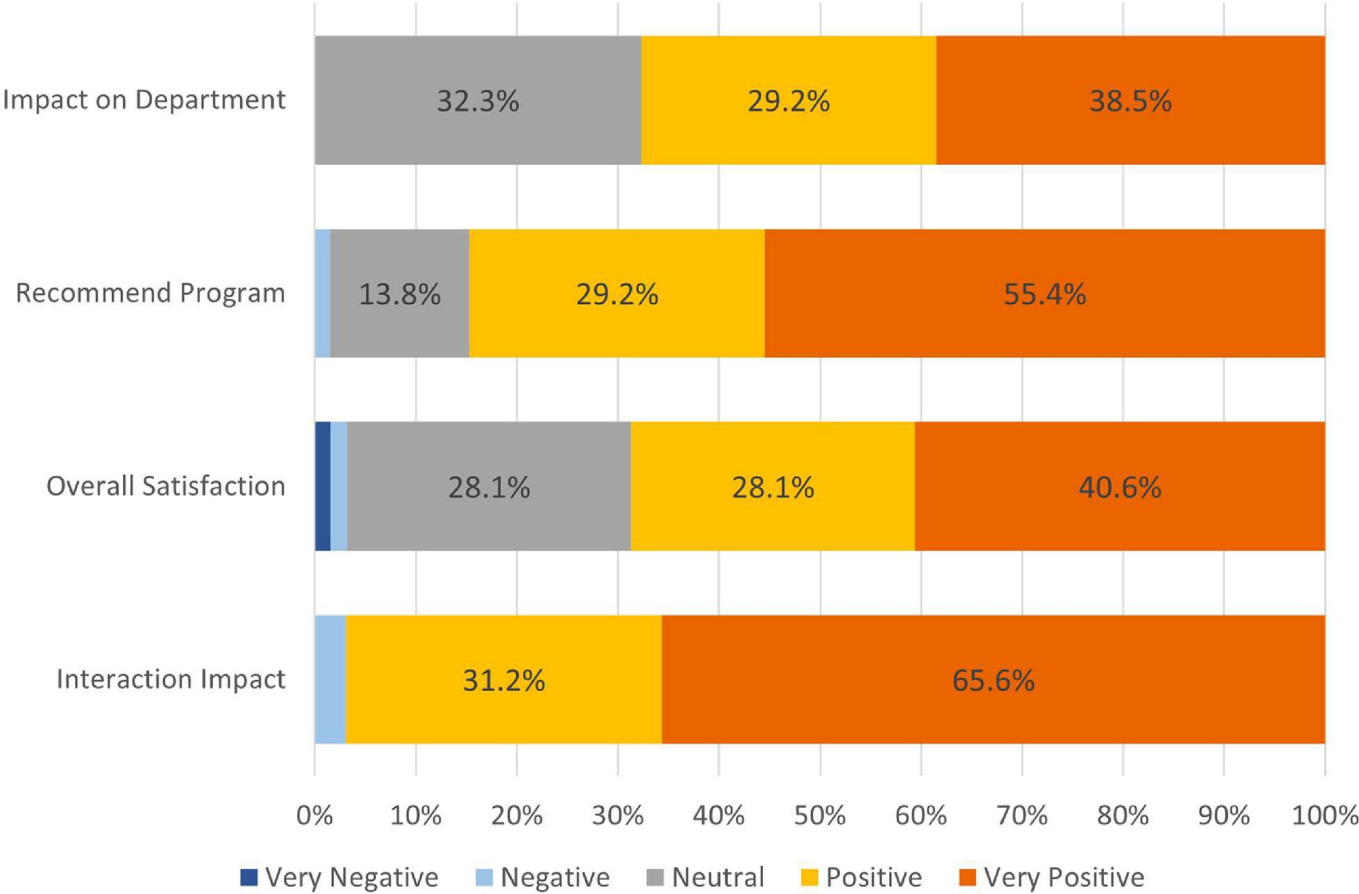
Programmatic feedback. 5-point Likert scale responses from the departmental survey displayed in 100% stacked bars among survey respondents who were aware of the POST program and responded to the question. n = 66 for “Impact on Department,” n = 65 for “Recommend Program” and n = 64 for “Overall Satisfaction.” “Interaction impact” refers to perceived helpfulness of a POST interaction by peer support recipient (n = 32).

For perceived effectiveness outcomes, we also considered results from the department-wide anonymous survey. Overall, physician respondents rated the program very favorably (Fig 4). The majority of the 32 respondents who had received peer support (31, 96.9%) found POST peer support helpful, with 27 (84.4%) reporting improvement in their well-being after a POST interaction, and 24 (75%) reporting it made them more comfortable talking about their work-related emotions.

We received open-ended responses from 42 (63.6%) of the 66 respondents who were aware of the program and our study team identified 8 broad themes of program benefits and potential barriers to use. We then divided these into perceived effectiveness outcomes (Table 2) and implementation outcomes (Table 3). Respondents who had used the program reported positive psychological impacts of the interaction, including perspective taking, increased personal resilience, and hopefulness. More broadly, respondents described systemic benefits of the POST program such as positive departmental cultural change and the normalization of conversations about clinical stressors. There was broad appreciation for the skill level of the peer supporters, who were described as good listeners, emotionally intelligent, unbiased, and providers of good advice.

**Table 2:** Major perceived effectiveness themes of Peer Outreach Support Team (POST) feedback. Major themes and representative quotes of surveyed physicians on their perceived impact of the POST program in open-ended response.

**Benefit—The Importance of Peers**	“Peers have a baseline level of understanding about the level of stress we face as well as an understanding of the specifics of our jobs and self-expectations, department culture, etc. This understanding allowed the supporter to meet me where I was without having to spend emotional effort and energy explaining the setting and background of the specific trauma we met to discuss.” “I think this is an incredible program. I have had some bad things happen in the last few years and have spoken with EAP and had my own therapist who I speak to on and off… The one constant has been my POST supporter. And in many ways, it has been the easiest therapeutic relationship to have because I don’t need to explain my work environment or the current struggles, especially when work is such a huge contributor to my mental health struggles.”
**Benefit—Mitigates Negative Emotions, Stigma, and Isolation**	“The interaction allowed me to share my thoughts and feelings in a consequence-less environment where judgment and/or next steps were not inevitable…. I felt less isolated, frustrated, and victimized afterwards.” “Felt less alone in my situation.”
**Benefit—Positive Psychological Impact**	“This program works on the micro level with individual physicians…in shifting our perceptions of mental health and the impact of the secondary trauma we experience in the field of medicine. It provides action and concrete methods for developing the emotional resilience that allow physicians to continue to practice with their full selves rather than have those physicians rely on maladaptive behaviors that ultimately erode their own health and diminish their capacity to provide exceptional care.” “I really enjoy the time with colleagues, and I feel it improves my personal wellness and [joy and meaning in medicine].”
**Benefit—Cultural Benefits**	“POST has provided a significant positive impact on our department. I think having the space and the support for peer support has helped many individuals and has helped our group morale overall.“ “POST has the potential to positively change the culture of medicine in general—can’t say enough positive endorsements of this program!”
**Benefit—Peer Supporters are Skilled**	“My supporter is an incredible listener. [POST] has also served to validate how I was feeling in terms of burnout and not wanting to work in the ER. Just to have a sounding board who truly understands what it is to feel the work pressures, low morale at work, negative interactions with colleagues/nurses/patients—it felt very validating to be understood. Also, her advice was excellent, and I think back to it a lot.” “My POST peer supporter was a good listener, helped me get out of my head, helped me visualize my career in a way I wasn’t able to in the mindset I was in at the point. It was invaluable to be able to have a frank discussion with someone who knew exactly what I was going through.”

**Table 3:** Major implementation themes of Peer Outreach Support Team (POST) feedback. Major themes and representative quotes of surveyed physicians on their perceived impact of the POST program in open-ended responses.

Benefit—Institutional Support	“I am extremely appreciative of the financial compensation because it means that my joy and meaning in medicine is valued by [the organization] and my wellness does not come at the expense of my personal time with my family.” “I find it very helpful that these sessions are recognized in a way as tangible as being able to bill. It makes me feel that my department/ physician group is really trying to invest in my wellness.”
Benefit—Accessibility, Proactive Outreach	“I appreciated that someone reached out to me from the program after a difficult situation.” “Just having someone reach out to acknowledge that I’d been involved in a difficult case felt comforting.” “Incredibly supportive and lowers the bar for providers to ask for/get help.”
Barrier—Time Constraints	“Would be great to have ‘office hours’ that we knew we could access people at certain times—contacting the POST leads and then asking to set up a time can be challenging with really short schedules.” “[It would be helpful to] schedule POST time for everyone, instead of having it be an ‘opt-in’ program.”

### Adoption

Of the 40 respondents to the department-wide survey who had had a peer supporter reach out to them, 32 (80%) accepted the support. Of 66 respondents with awareness of the program, 55 (83.3%) would recommend the program to another department.

Over the three years of study, 11 departments had voluntarily adopted the POST program. This represents approximately 530 physicians, or approximately 60% of all physicians in the 2-hospital system. By September of 2022, 3 years and 3 months after initial launch, 7 additional hospitals had successfully launched the POST program, with more expressing interest.

### Implementation

#### POST program implementation

Implementation of the POST program remained a grassroots approach, relying on word of mouth, leadership promotion, and interest and requests from local departments to start a program. This contributed to local momentum and greater ease of implementation. Small groups of peer supporters were trained at a time, maintaining an atmosphere of participation and interaction. Spread was a natural and gradual process spurred by increasing acceptance of peer support and cultural change. Facilitators as identified in the PRISM framework (Fig 1) included strong leadership support, departmental adaptations to meet specific needs, peer matching, institutional and financial support, long term vision, and a high demand and expressed need for the program (high levels of burnout in healthcare). Barriers included a hospital culture of toughness, busy work schedules, and at times, a level of burnout that prevented providers from accessing help.

Department-wide survey respondents reported high rates of awareness of and satisfaction with the program (Fig 4). A majority of physician respondents in surveyed departments were aware of the POST program (66 out of 75, 88%). In open-ended survey responses, several positive implementation themes emerged, including the positive impact and symbolism of institutional support, and the accessibility of POST with its ease of access and its proactive nature of referrals (Table 3).

Reported barriers to POST acceptance identified in the survey included lack of time to schedule a POST interaction (36 out of 66 respondents, 54.5%), lack of perceived need (16, 24.2%), privacy concerns (12, 18.2%), not being comfortable talking about emotions with colleagues (12, 18.2%), and medical-legal concerns (2, 3%). Of survey physicians who rated their overall satisfaction with the program as neutral (18 out of 64 respondents with awareness of the program, 28.1%) or negatively (2, 3.1%), only 3 (15%) had had a POST interaction, whereas of those who rated their overall satisfaction as very satisfied (26, 40.6%) or satisfied (18, 28.1%), 30 (68.2%) had had a POST interaction. Difficulty accessing the program with time constraints was also identified as a barrier in open-ended responses (Table 3).

#### POST initial training implementation

Of 37 trainee feedback survey respondents, 100% described the training as good or excellent on a 5-point bimodal Likert scale, with high perceptions of confidence and satisfaction with the training (Fig 3). We found 32 (86.5%) of respondents evaluated the virtual platform positively. In open-ended responses, 24 (64.9%) of respondents cited role-play activities and 7 (18.9%) cited resource review as the most helpful components of the training, while adding more difficult practice scenarios and lengthening the training were the most often cited suggestions for improvement (5, 13.5% and 4, 10.8% respectively, S3 File).

### Maintenance

The number of successful interactions per year increased over the 3-year study period across all active departments, from 73 in the first year, to 109 in the 2nd year, to 124 in the third year of the study period. The average number of interactions per month increased from 6.1 in the first year of the program to 10.3 in its 3rd year.

The program was able to achieve and maintain the target ratio of 1:10 peer supporters to physician staff in all departments (S2 File). Of the initial 59 peer supporters trained, 50 (84.7%) are still active as peer supporters as of December 2022. All departments that implemented a POST program continue to have active programs (100%), and the POST program continues to spread to additional departments and hospitals across the region, with 7 additional hospitals within KPNC having initiated teams by September 2022 and more with plans to adopt the program. POST is recognized as a regional initiative with institutional support on local and regional levels and a growing regional professional community.

## Discussion

We describe the development and key guiding principles of the POST program, a novel physician peer support program, and its implementation in our health system. Applying the PRISM framework, we identify important contextual factors that impacted the implementation of the program in a 2-hospital system. The success of the program can be seen in its substantial reach, perceived effectiveness, physician adoption, and continued spread to additional departments and hospitals. The use of PRISM and consideration of RE-AIM outcomes allows for important conclusions which may guide spread and local adaptations in future sites.

Several contextual factors were key determinants in POST implementation. Key facilitators included optimal peer matching, institutional support, and financial support. The survey data confirmed that providers valued institutional and financial support as a positive implementation theme. Respondents also noted the importance of peers, allowing more profound understanding and connection. The departmental structure allowed true peer-to-peer matching, an aspect well-demonstrated in the literature as beneficial and most widely accepted by physicians [19]. Barriers to implementation and use of the program included busy work schedules, burnout, and a hospital culture of toughness. We found that physicians reported a lack of time most often as a barrier to accessing the program. This warrants further inquiry and suggests that more tailored institutional time protection may be an important consideration in establishing successful peer support programs. Additional barriers included a lack of perceived need, privacy concerns, and discomfort with talking about emotions. These identified facilitators and barriers should be considered in any application of a peer support program for physicians.

Earlier models of peer support programs in healthcare reported lower physician reach, with fewer referrals and more limited program spread [20–23]. Shapiro and Galowitz argued for a third-party referral system to better reach clinicians based on their own experience implementing a peer support program [23], yet to our knowledge, prior studies have not studied the use of a third-party referral system directly. In feedback surveys, we found widespread reach of the program with 48.5% of respondents having had a peer support interaction, and 306 successful interactions in 3 years across the active departments. In open-ended responses, physicians identified components of the third-party referral system, including proactive outreach and taking the onus off the suffering provider, as strongly positive implementation factors. We attribute the POST program’s greater engagement of physicians compared to previous studies to several unique factors, paramount among them the robust third-party referral system.

Our survey data shows that physicians felt the POST program has been effective. As far as we know, this is the first evaluation of a peer support program to incorporate and report end-user feedback, which allowed individual and culture-level perceived effectiveness considerations. Physician recipients of peer support reported increases in well-being, mitigation of negative emotions and stigma, and beneficial cultural shifts within their departments. Respondents, even those who had not used the program, felt having the program available helped bring positive cultural shifts toward greater acceptance of self-care and mental health practices in their departments. Our findings suggest both individual and system-level benefits of an effective physician peer support program.

We found that the POST program was adopted broadly across the 2-hospital system and has since successfully spread to other hospitals across the region, with high retention of peer supporters trained. A majority of respondents in active departments would recommend the program to another department, and this lends itself to further spread, as we rely on word of mouth and requests to bring the program to new locations. Acceptance was maintained over time, with annual rates of peer support interactions increasing each year over the 3-year period. These findings together suggest a successful bottom-up approach for rollout of a peer support program, relying on successful pilot departments, word of mouth, and gradual spread to departments and medical centers who are ready to adopt the program.

Access to skilled peer supporters was identified as a key facilitator of the program. Positive reviews of the peer supporter training suggest that effective training enabled peer supporters to feel confident supporting their peers. Many peer supporters valued hands-on training and practice, including role-play and breakout sessions. Most trainees rated the virtual platform very highly, a potential leverage point to ensure consistent training standards and efficient, effective spread. We suspect there is an important relationship between the value of the training and the positive impact and perception of the program, and an emphasis on robust training may increase the success of any peer support initiative.

While the POST program had positive impacts across multiple departments, EM had the highest number of referrals. This may be due to a high number of critical cases, a greater burden on front-line providers during the COVID-19 pandemic, and higher rates of burnout in this specialty [8]. The more team-based practice setting in EM may also be conducive to third-party referrals. Clinic-based departments had fewer support interactions, with survey respondents citing time constraints as the main barrier to access. More in-depth qualitative research within departments could help test these hypotheses and provide further insight into how peer support programs could be adapted across contexts. Considering these findings, we encourage tailoring the time and referral support to specific specialty practice patterns to accommodate the unique needs of clinic versus hospital-based physicians.

### Limitations

The departmental survey was administered via email with only 1 reminder one week later. Limitations to response include lack of financial incentives, busy schedules, and full email inboxes. The number of physicians who opened the email and those who were on PTO or leave is not known, and these physicians were likely missed. Given these factors, we believe the response rate still provides a broad and diverse cross-section of the population of interest allowing meaningful conclusions. A future study may put into place financial incentives or a more direct survey measure to garner a higher response rate.

This study considered perceived effectiveness for trained peer supporters and physician recipients of peer support. While meaningful measured outcomes included confidence and tool acquisition for peer supporters, positive psychological impacts of peer support, changes in wellbeing, and perceived cultural benefits, we did not measure direct effectiveness outcomes including direct quantitative measures of burnout, sick leave, extended time away from work, physician turnover, nor direct measures of departmental culture and psychological safety. The findings suggest future studies are needed that include rigorous direct effectiveness outcomes.

Our study period included a 3-year period. We believe this allowed effective evaluation of the program’s reach, adoption, maintenance, and perceived cultural and individual well-being effects. A future study could consider a longer time period to measure and draw conclusions regarding the long-term effect of a peer support program on a living and evolving healthcare organization.

This study was conducted in a unique clinical environment, a large integrated healthcare delivery system providing comprehensive inpatient, ED, and ambulatory care for over 4 million people, staffed by a multi-specialty group of physicians. The results from this study are not universally generalizable to other healthcare systems or physician groups. Future studies of peer support programs in a variety of healthcare models are needed to delineate the generalizability of these findings.

### Conclusions

Physician burnout impacts physician health, the effective functioning of our healthcare systems, and patient outcomes. We found that POST, a physician-focused peer support program, had widespread reach and a positive effect on perceived physician well-being and departmental culture. Several components of the POST program, including its physician and department-level focus, robust institutional support, third-party referrals, and value placed on effective peer supporter training, were identified as facilitators, and likely contributed to the program’s reach and perceived effectiveness. Our findings can inform and guide other healthcare systems striving to establish peer support initiatives to improve physician well-being.

## Supporting information

S1 Appendix (DOCX)

S1 File (XLSX)

S2 File (XLSX)

S3 File (XLSX)
