# Supplementary material for: Implementation and effectiveness of a physician-focused peer support program
Source: PLoS One. 2023 Nov 1;18(11):e0292917. doi: 10.1371/journal.pone.0292917 (PMC10619771; doi:10.1371/journal.pone.0292917)
Supplement: S1 Appendix — (DOCX) [file pone.0292917.s001.docx]

**Supplementary Appendix**:

**Table of Contents:**

Supplemental Figures S1-3 --------------------------------------- Pages 1-3

Survey 1 ---------------------------------------------------------------- Pages 3-5

Survey 2 ---------------------------------------------------------------- Pages 5-10


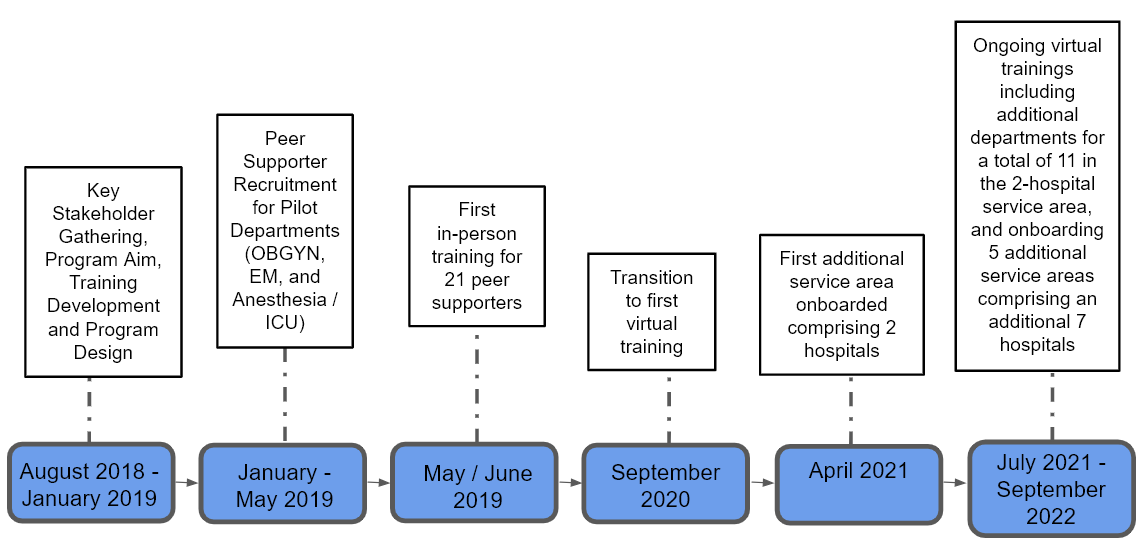


**Supplemental Figure S1: Timeline for Peer Outreach Support Team Creation and Launch**. Timeline includes genesis, initial recruitment and training, transition to virtual training, and ongoing spread within the 2-hospital system and to other service areas. OBGYN, Obstetrics and Gynecology; EM, Emergency Medicine. ICU, Intensive Care Unit.


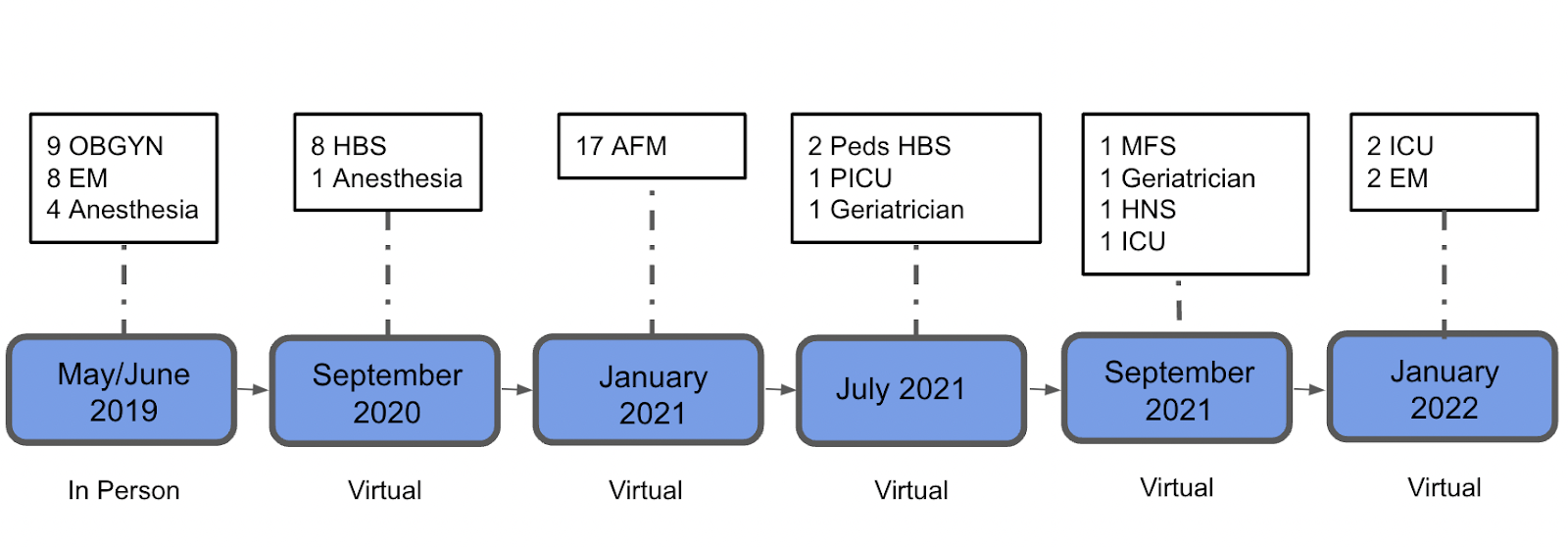


**Supplemental Figure S2: Timeline for physicians trained.** Number of physicians trained by date in the 2-hospital service area of focus. Displayed above date is number of physicians and specialty; displayed below date is whether peer supporters received in person or virtual training. OBGYN, Obstetrics and Gynecology; HBS, Hospital Based Services (Hospitalists); EM, Emergency Medicine; PICU, Pediatric Intensive Care Unit; HNS, Head and Neck Surgery; MFS, Maxillofacial Surgery; ICU, Intensive Care Unit; Peds, Pediatrics


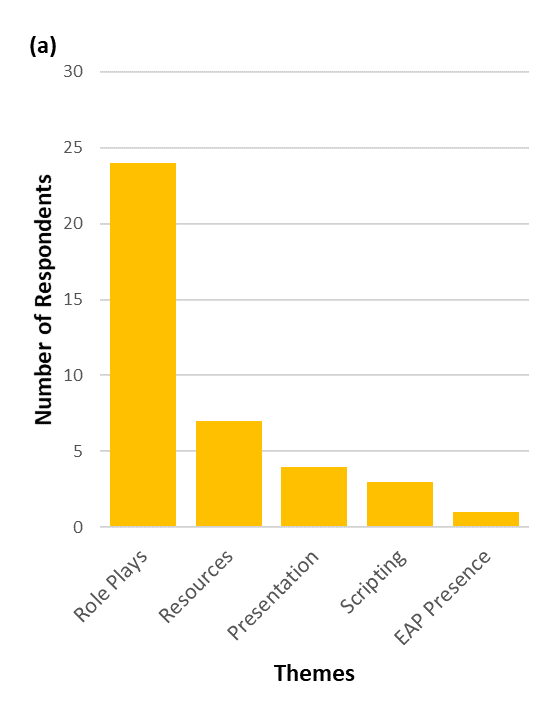

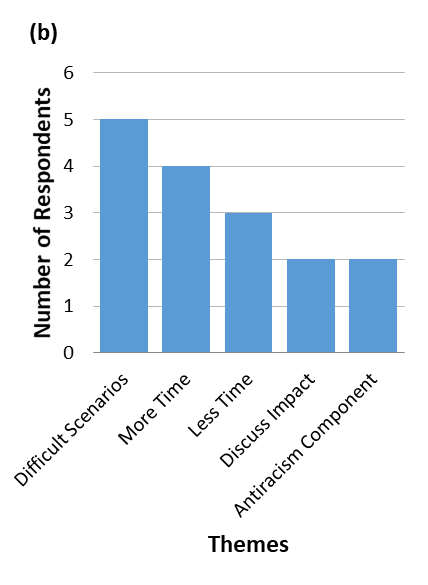


**Supplemental Figure S3**: **Thematic feedback for Peer Outreach Support Team (POST) training.** Number of respondents who cited these categories in open-ended response as (a) existing components most beneficial to the training and (b) ways the training could be improved. “Resources” refers to review of existing mental health resources available to physicians. “Presentation” refers to the training materials, including PowerPoint slides, training content, and delivery. “Scripting” refers to scripting and phrases provided prior to practice sessions. “EAP (Employee Assistance Program) Presence” refers to the participation of an EAP consultant. “Difficult Scenarios” refers to adding more challenging mock scenarios during practice sessions. “Discuss Impact” refers to the desire for more discussion around results of the program in other departments. “Antiracism Component” refers to the desire to include training around anti-racism in peer support. Respondents could cite more than 1 theme and each response category was counted in figure above. Number of survey respondents = 37.

**Survey 1:** **POST Training Feedback Survey.** This was an anonymous survey administered via Microsoft Forms. It was distributed via a link in an email inviting participation.

1. Overall, you would rate this training:

- 1. Excellent
  2. Good
  3. Neutral
  4. Poor
  5. Very Poor

2. This training focuses largely on formal avenues of support. An additional goal of the POST program is to shift culture towards mutual support in all of our interactions. Did this training give you a framework and tools that you would use in your everyday practice and collegial interactions outside of POST?

- 1. Yes
  2. Maybe
  3. No

3. Based on this course, I can navigate the resources available to my colleagues after a difficult or adverse event.

- 1. Strongly Agree
  2. Agree
  3. Neutral
  4. Disagree
  5. Strongly Disagree

4. What is still unclear? (Open ended response)

5. I understand my role as a peer supporter in the POST program after this training.

- 1. Extremely Well
  2. Somewhat Well
  3. Neutral
  4. Poorly
  5. Extremely Poorly

6. What is still unclear? (Open ended response)

7. How well do you understand the referral processes for POST in your department?

- 1. Extremely Well
  2. Somewhat Well
  3. Neutral
  4. Poorly
  5. Extremely Poorly

8. What is still unclear? (Open ended response)

9. What part of this training did you get the most out of? (Open ended response)

10. What are ways this training could be improved? (Open ended response)

11. Understanding that Microsoft Teams has inherent limitations, Teams as a platform for this training was:

- 1. Great
  2. Good
  3. Neutral
  4. Poor
  5. Very Poor

12. Any other feedback specifically on the virtual platform used for this training?

13. Please add anything else you want us to know, ideas you have for improving or changing the training, or any other feedback here. (Open ended response)

**Survey 2**: **Departmental feedback survey.** A department-wide anonymous survey was sent to all attending physicians via email in the longest-running departments with active peer supporter teams, including Obstetrics and Gynecology, Emergency Medicine, Hospitalists, the Intensive Care Unit, and Anesthesia. This was an anonymous survey administered via Microsoft Forms. It was distributed via a link in an email inviting participation.

1. What is your department?

- 1. Emergency Medicine
  2. Anesthesia
  3. OBGYN
  4. Hospitalists
  5. ICU

2. What is your age?

- 1. 25-30
  2. 30-35
  3. 35-40
  4. 40-45
  5. 45-50
  6. 50-55
  7. 55-60
  8. 60-65
  9. 65-70
  10. 70+

3. To which gender identity do you most identify?

- 1. Man
  2. Woman
  3. Non-binary
  4. Prefer not to say
  5. Other (fill in)

4. How many years have you been in practice (post-residency)?

- 1. 0-5
  2. 5-10
  3. 10-20
  4. 20-30
  5. 30+

5. Are you aware of the POST Peer Support Program? (If no, survey ends)

- 1. Yes
  2. No
  3. Maybe

6. Have you made a referral for someone else to the POST program?

- 1. Yes
  2. No

7. Have you made a self-referral to the POST program?

- 1. Yes
  2. No

8. What impact has the POST program had on your department?

- 1. Very Negative Impact
  2. Somewhat Negative Impact
  3. Neutral
  4. Somewhat Positive Impact
  5. Very Positive Impact

9. Overall, how satisfied are you with the POST program?

- 1. Very unsatisfied
  2. Unsatisfied
  3. Neutral
  4. Satisfied
  5. Very Satisfied

10. Is there anything you would like to tell us about your perception of the program overall? (Open ended response)

11. Would you recommend this program to other departments or physicians?

- 1. Yes, would highly recommend
  2. Yes, would recommend
  3. Neutral
  4. No, would not recommend

12. What potential barriers may limit your use of the program or your acceptance of POST peer support? Select all that apply.

- 1. Lack of time
  2. Privacy concerns
  3. Med-Legal concerns
  4. Don’t feel like I need it
  5. Not comfortable talking about my feelings with colleagues
  6. Other (fill in)

13. What might make you feel more comfortable accessing the program? (Open ended response)

14. Burnout is a syndrome of depersonalization, negative attitudes, and emotional/physical/psychological exhaustion. How would you rate your own level of burnout?

- 1. Very High
  2. High
  3. Moderate
  4. Low
  5. Very Low

15. Have you ever had a POST peer support interaction?

- 1. Yes
  2. No, a peer supporter has never reached out to me
  3. No, a peer supporter has reached out to me but I declined

16. How many peer support interactions have you had? (If you are a POST peer supporter, please only count the interactions where you were the physician receiving support)

- 1. 1
  2. 2
  3. 3
  4. 4
  5. 5+

17. How Helpful was the peer support interaction(s)?

- 1. Very Helpful
  2. Helpful
  3. Somewhat Helpful
  4. Not at all Helpful
  5. Negatively Impacted Me

18. Can you please share why you found your interaction with a POST peer supporter helpful or unhelpful? (Open ended response)

19. What change if any did you notice in your emotional wellbeing after your POST peer support interaction(s)?

- 1. Significant Improvement
  2. Some Improvement
  3. No Change
  4. Some Decline
  5. Significant Decline

20. Did your POST peer supporter help you access additional support resources such as EAP, Physician Well Being Committee, or others?

- 1. Yes
  2. No

21. Did your POST interaction change your comfort with talking about your work-related feelings/emotions in general?

- 1. It made me much more comfortable talking about my work-related feelings/emotions in general
  2. It made me somewhat more comfortable talking about my work-related feelings/emotions in general
  3. It did not change my comfort talking about my work-related feelings/emotions in general
  4. It made me less comfortable talking about my work-related feelings/emotions in general

22. Is there anything else you can share about your POST interaction? (Open ended response)

23. If there is anything else you would like to add about POST as a program, any POST interaction, or any other feedback or thoughts, please share here. (Open ended response)
